# Amino Acid Polymorphisms Within the Entire HCV NS5A Region in Estonian Chronic HCV 1b Patients With Different Treatment Response

**DOI:** 10.5812/hepatmon.14481

**Published:** 2013-12-14

**Authors:** Tatiana Kuznetsova, Tatjana Tallo, Vadim Brjalin, Irina Reshetnjak, Riina Salupere, Ljudmilla Priimagi, Olga Katargina, Maria Smirnova, Juris Jansons, Valentina Tefanova

**Affiliations:** 1 Department of Virology, National Institute for Health Development, Tallinn, Estonia; 2 Department of Diagnostics and Vaccine, Unit for Molecular Typing, Swedish Institute for Communicable Disease Control, Stockholm, Sweden; 3 Department of Internal Medicine, West-Tallinn Central Hospital, Tallinn, Estonia; 4 Department of Internal Medicine, University of Tartu, Tartu, Estonia; 5 Department of Hemorrhagic Fevers and Pilot Projects, Federal State Budgetary Institution “Chumakov Institute of Poliomyelitis and Viral Encephalitides” of RAMS, Moscow, Russia; 6 Protein Engineering Department, Latvian Biomedical Research and Study Centre, Riga, Latvia

**Keywords:** Estonia, Hepatitis C, Ribavirin, Therapy

## Abstract

**Background:**

A substantial proportion of hepatitis C virus (HCV)-1b infected patients do not response to pegylated interferon-α plus ribavirin (PegIFNα/RBV) combination therapy that was partially associated with mutations in the non-structural 5A (NS5A) protein.

**Objectives:**

Analysis of NS5A polymorphisms in HCV genotype 1b pre-treatment serum samples from Estonian patients and their effect on the treatment response.

**Patients and Methods:**

Twenty-nine complete NS5A sequences obtained from patients with chronic HCV-1b infection who had received combined therapy with PegIFNα-2a/RBV were analyzed and compared with the prototype strain HCV-J. Twelve patients achieved a sustained virological response (SVR), 15 were non-SVR and 2 patients stopped treatment because of side effects.

**Results:**

No significant difference in total number of amino acid mutations was observed between isolates from SVR and non-SVR patients in any known regions of the NS5A protein. However, specific amino acid substitutions at positions 1989 and 2283 correlated significantly with SVR, mutations at positions 1979, 2107, 2171 and 2382 were associated with non-response to treatment and amino acid substitution at position 2319 was observed in relapsers. At phylogenetic analysis, NS5A nucleotide sequences have been subdivided into four groups characterized by the different treatment response. Twenty-four novel nucleotide polymorphisms and 11 novel amino acid polymorphisms were identified based on the phylogenetic tree topology.

**Conclusions:**

Specific amino acid substitutions correlating with the treatment response were found. Polymorphisms revealed by phylogenetic analysis may define the signature patterns for treatment susceptible and treatment resistant strains prevalent in Estonia.

## 1. Background

Hepatitis C virus (HCV) is a major cause of chronic hepatitis, cirrhosis and hepatocellular carcinoma (1). With an approximate estimate of 3% of the world population infected with HCV and the lack of a preventive vaccine, HCV infection remains a serious burden to public health worldwide (2).

Despite the development of new directly-acting antivirals (DAA) for treatment of HCV (3), a combination therapy with pegylated interferon-alpha plus ribavirin (PegIFNα/RBV) still remains the standard of care for chronic hepatitis C (CHC) (4). However, this therapy is costly, requiring long-term follow-up and involving severe side effects, and is not effective for all patients (5).

The criterion for evaluation of the efficacy of therapy is sustained virological response (SVR) (6). Additionally, early virological response (EVR) is another reliable marker determining the duration and outcomes of treatment for CHC patients (7).

The rate of SVR to combination therapy is dependent on the HCV genotype. It is well established that genotypes 1 and 4 are more resistant to IFN-based therapy than genotypes 2 and 3 (8). Subtype 1b is the most prevalent genotype in Estonia (9). 

Amino acid (aa) micro-polymorphisms within non-structural 5A protein (NS5A) have been the main focus in a several of studies (10, 11). These polymorphisms are described in terms of the number of mutations which make the sequence divergent from the HCV-J prototype, subtype 1b (12). The prognosis for therapy outcome is suggested to be as good as many differences from the prototype are found in the NS5A protein of the viral isolate.

Mutations within the IFN sensitivity-determining region (ISDR; aa 2209-2248), the protein kinase-binding domain (PKRBD; aa 2209-2274), the variable region 3 (V3; aa 2356-2379), and the interferon/ribavirin resistance-determining region (IRRDR; aa 2334-2379) in the NS5A protein of HCV have been correlated with the IFN-based therapy response (12-15). 

However, controversial data have also been presented regarding the correlation of NS5A heterogeneity with treatment response, particularly in populations from Europe (16, 17) and the United States (15).

## 2. Objectives

In the present study, we investigated mutations in the entire NS5A protein in pre-treatment serum samples from Estonian patients with chronic HCV genotype 1b infection, and analyzed their effect on the PegIFNα/RBV treatment response. We also examined if there is any correlation between the NS5A sequence variations and EVR.

## 3. Patients and Methods

### 3.1. Patients

Twenty nine treatment-naive patients with chronic hepatitis C started combination therapy with PegIFNα/RBV at the Outpatient Clinic of West-Tallinn Central Hospital, Tallinn, Estonia, between April 2006 and June 2010 were subjects for our single-centre study. The diagnosis of CHC was based on the presence of HCV RNA in the sera, histologically verified fibrosis stage, degree of inflammatory activity and clinical follow-up. The enrollment and further treatment of patients were conducted according to the National Guidelines on the treatment of CHC. 

The treatment exclusion criteria were age < 18 and > 63 years, chronic alcohol intake, decompensated cirrhosis, current injection drug use and depression.

All patients received Peg-IFN α-2a at a standard dosage of 180 μg/week and RBV at a dosage of 1,200 mg/day or 1,000 mg/day, depending on body weight (above or below 75 kg) for 48 weeks. An SVR was defined as undetectable serum HCV RNA at the end of treatment and at post-treatment week 24. Complete EVR (cEVR) was defined as undetectable HCV RNA at week 12. Relapse (RL) was defined as undetectable HCV RNA at the end of treatment and reappearance within 24 weeks after the end of therapy. Non-response (NR) was defined as detectable HCV RNA during and at the end of treatment. Patients who stopped treatment because of side effects were denoted as ST. 

This study was approved by the Tallinn Medical Research Ethics Committee, Code nr.1130.

Written informed consent for use of clinical data and serum samples was obtained from each participant prior to the study.

### 3.2. Detection of HCV RNA 

Quantitative serum HCV RNA levels at baseline and at weeks 4, 12, 24 and 48, and 24 weeks after treatment were analyzed by the quantitative PCR assay (COBAS® AmpliPrep/COBAS® TaqMan HCV test, a lower limit of detection of 15 IU/mL, Roche, Branchburg). 

### 3.3. HCV RNA Extraction and cDNA Synthesis

HCV RNA was extracted from 200 µl of pre-treatment serum using TriPure isolation reagent (Roche) according to the manufacturer’s recommendations. The extracted RNA was reverse transcribed and amplified for full-length NS5A using SuperScript III reverse transcriptase (Invitrogen) and random primers (Fermentas) according to the manufacturer’s instructions.

### 3.4. Entire NS5A Amplification, Sequencing and Phylogenetic Analysis

The entire NS5A gene (1341 nucleotides; nt) was amplified with external primers 5’-ATGAACCGGCTGATAGCGTT-3’ and 5’-CTCCTTGAGCACGTCCCGGT-3’ and internal primers 5’-TCCCCCACGCACTATGTGCC-3’and 5’-CGGTARTGGTCGTCCAGGAC-3’. PCR products were sequenced directly with PCR internal primers used for amplification and primer 5’-ATTCCAGGTCGGGCTCAA-3’. The obtained sequences were edited with BioEdit v7.0.8.0 software and aligned with the NS5A region of HCV-J prototype for genotype 1b (GenBank accession number D90208) with the ClustalW multiple alignment utility. Phylogenetic analysis was carried out using the MEGA software (http://www.megasoftware.net) using the Kimura-2 parameter model. The phylogenetic tree was constructed by the maximum likelihood (ML) method using the MEGA 5.2 program. Bootstrap analysis was performed on 1000 replicates. 

All sequences reported herein were deposited in the NCBI GenBank nucleotide sequence databases with accession numbers JX022751-JX022779.

### 3.5. Statistical Analysis 

Comparisons between groups were made by the Student’s t-test or the Mann-Whitney test for quantitative variables and the chi-square or Fisher’s exact test for categorial variables. The quantitative variables were expressed as mean ± SD. The correlation between variables was estimated by Pearson’s or Spearman’s coefficients of correlation. A two-tailed P value of less than 0.05 was considered statistically significant. Confidence intervals (CI) are 95%. 

## 4. Results

### 4.1. Baseline Characteristics of Studied Patients and Treatment Response

Among the patients enrolled in the study, 18 were males and 11 were females. Mean age was 41.5 ± 12.7 years; age range 19-60 years. At baseline, the viral load in 26 (89.7%) of the patients was higher than 600 000 IU/mL. Histologically, 21 (72.3%) of the patients had fibrosis (F) scores of 0 - 2. Twelve patients (41.4%) achieved SVR, whereas 15 patients (51.7%) failed to achieve SVR and were referred to as non-SVR. Of these, eight (53.3%) were NR and seven (46.7%) were RL patients. All SVR and RL patients but only 2 of the 8 NR patients achieved cEVR. Two patients stopped treatment because of adverse side effects. The baseline characteristics of the patients and the response to therapy are summarized in Table 1. 

**Table 1.** Comparison of Baseline Features in Patients with SVR and non-SVR^a^

Characteristics	SVR (n = 12)	Non-SVR (n = 15)	P value
**Age, Mean ± SD, y**	39.4 ± 12.7^b^	45.6 ± 9.5	0.158
**Gender, Male/Female**	6/6	10/5	0.452
**Viral load, × 10** ^**3**^ ** IU/ml**	1217 ± 699	1801 ± 1220	0.317
**Fibrosis stage, F0-2:3-4 ** ^**с**^	11:1	10:5	0.999
**ALT ** ^**d**^ **, IU/L (N ≤ 42)**	123.8 ± 151.7	73.9 ± 54.2	0.246
**GGT ** ^**d**^ **, IU/L (N ≤ 61)**	58.9 ± 55.2	105 ± 120.2	0.295

^a^Data excluded for 2 patients who stopped treatment

^b^ The data are presented as mean values ± standard deviation

^с^ Liver histology was graded according to the Metavir scoring system: F0, no fibrosis; F1, portal fibrosis without septa; F2, portal fibrosis with rare septa; F3, numerous septa without cirrhosis; F4, cirrhosis.

^d^Abbreviations: ALT, alanine aminotranferase; GGT, gamma-glutamyl transferase

The SVR and non-SVR patients did not differ significantly in terms of age, gender, alanine aminotranferase (ALT) and gamma-glutamyl transferase (GGT) levels, viral load, and stage of fibrosis. However, patients with SVR showed trends toward being younger and having higher ALT and lower GGT levels than those with non-SVR, although these differences were not statistically significant (Table 1). 

### 4.2. NS5A Polymorphisms and Response to Combination Therapy

Five out of the 29 studied patients (17.2%) were infected with wild-type ISDR IFN-resistant strains (0 mutations), and 24/29 (82.8%) of the patients were infected with intermediate type ISDR strains (1-3 aa mutations). None of the patients were infected with a mutant type strain (≥ 4 mutations).

**Figure 1.**
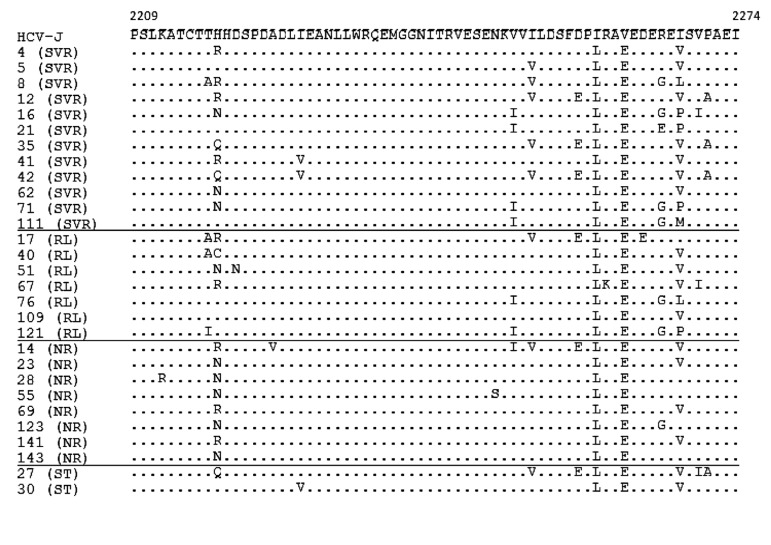
Amino Acid Sequence of the PKRBD Region (2209–2274) in Genotype 1b-infected Patients Each sequence was compared with HCV-J. The sequences were clustered according to the patient’s treatment response. The positions of the first and last amino acids of the PKRBD in the HCV polyprotein are indicated over the HCV-J sequence. Each patient is designated by a number and response to treatment. Abbreviations: SVR, sustained virological response; RL, relapse; NR, non-response; ST, stopped treatment.

**Figure 2.**
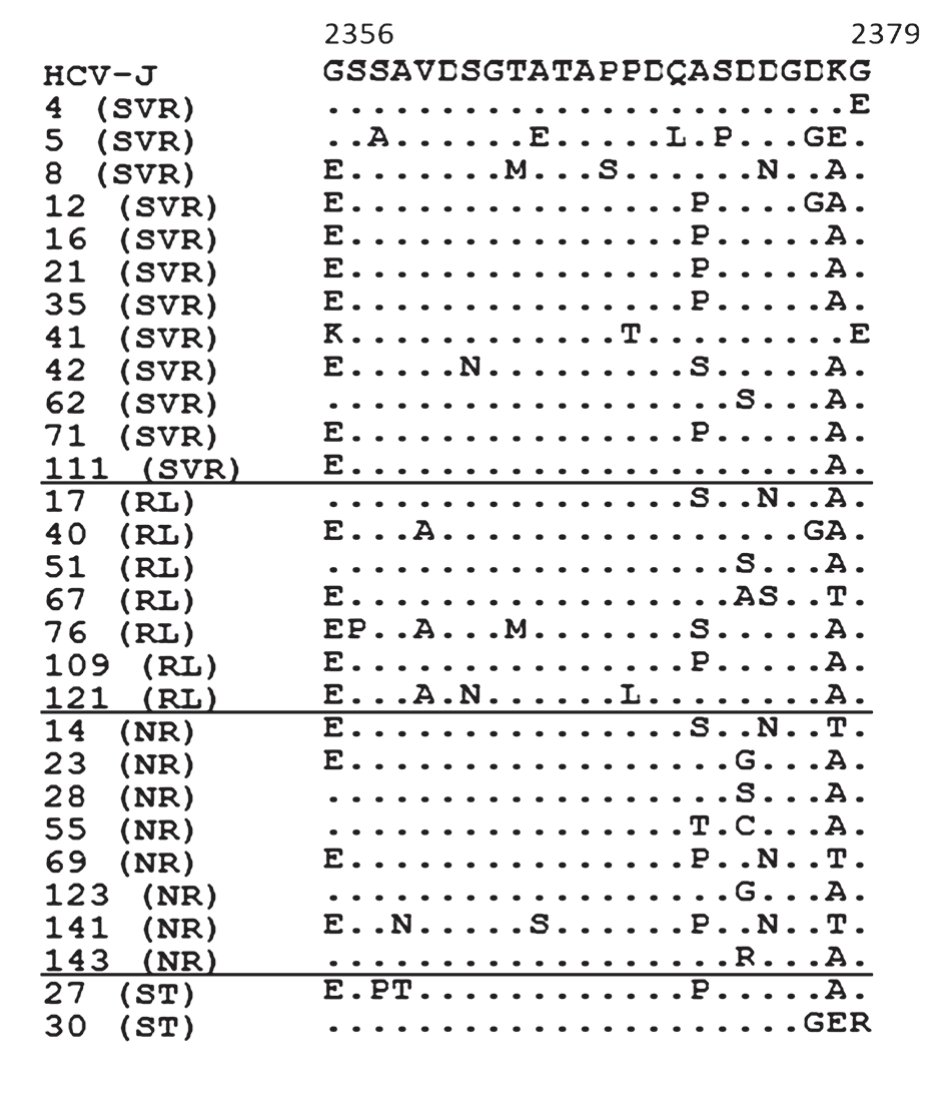
Amino Acid Sequence of the V3 Region (2356-2379) in Genotype 1b-infected Patients Each sequence was compared with HCV-J. The sequences were clustered according to the patient’s treatment response. The positions of the first and last amino acids of the V3 domain in the HCV polyprotein are indicated over the HCV-J sequence. Each patient is designated by a number and response to treatment. Abbreviations: SVR, sustained virological response; RL, relapse; NR, non-response; ST, stopped treatment.

No statistically significant associations were observed between treatment response and mean numbers of aa mutations either in the ISDR, PKRBD, V3 or IRRDR regions, or in the NS5A gene overall, between SVR and non-SVR patients including those with cEVR and non-EVR (Figure 1, 2). 

### 4.3. Specific Mutations Within the NS5A Protein and Outcome

**Figure 3.**
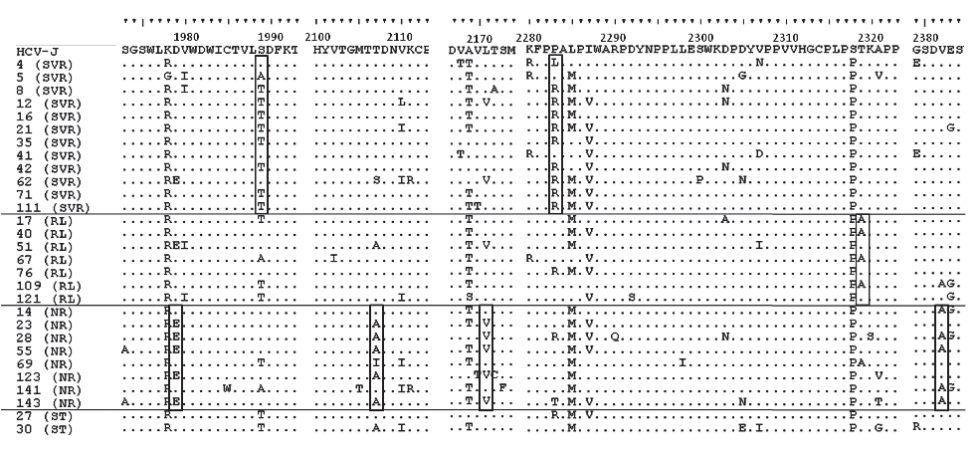
Essential Amino Acid Polymorphisms in the NS5A Region in Viral Isolates from Patients with Different Susceptibilities to Therapy Each sequence was compared with HCV-J. The sequences were clustered according to the patient’s treatment response. The positions of amino acids in the HCV polyprotein are indicated over the HCV-J sequence. Each patient is designated by a number and response to treatment. Essential polymorphisms are shown in boxes. Abbreviations: SVR, sustained virological response; RL, relapse; NR, non-response; ST, stopped treatm.

Pre-treatment aa sequence variants within the NS5A protein were compared for SVR and non-SVR patients (Figure 3). 

Sequence analysis of the cytoplasmic retention signal (CRS; aa 1973-1999) at codon 1979 revealed D in 11/12 (91.7%) of SVR patients, instead of E in 5/8 (62.5%) of NR patients, P = 0.018 (Figure 3). T at codon 1989 was found in 8/12 (66.7%) of patients with SVR, instead of S in 6/8 (75%) of NR patients, P=0.028. Three out of the 12 patients (25%) with SVR, 1/7 (14.3%) of RL patients and 1/2 (50%) of ST patients had V instead I at codon 2333 in the nuclear localization signal (NLS; aa 2326-2334), but any correlation of this mutation with therapy outcome was not statistically reliable. 

For 5/8 (62.5%) of NR patients there were identified mutations in codon 2107 (T to A), P = 0.004, in codon 2382 (V to A), P=0.004, and the less statistically significant mutation L to V in codon 2171, P = 0.06 (Figure 3). 

R at codon 2283 was present in 9/12 (75%) of patients with SVR, and was significantly correlated with viral loads below 1,000,000 IU/mL (CI 35.4-84.8%; P < 0.05), 7/11), instead of P in 12/15 (80%) of non-SVR patients, which correlated significantly with viral loads higher than 1,000,000 IU/mL, (CI 44.4-85.7%; P < 0.05), (Figure 3). 

T at codon 2319 was found in 12/12 (100%) of SVR patients, instead of A in 4/7 (57.1%) of relapse patients, P = 0.009. T was also found in three of the remaining relapsers. 

The aa substitutions throughout the NS5A protein in the two ST patients were in general similar to those in SVR patients (Figure 3). 

### 4.4. Phylogenetic Analysis 

**Figure 4.**
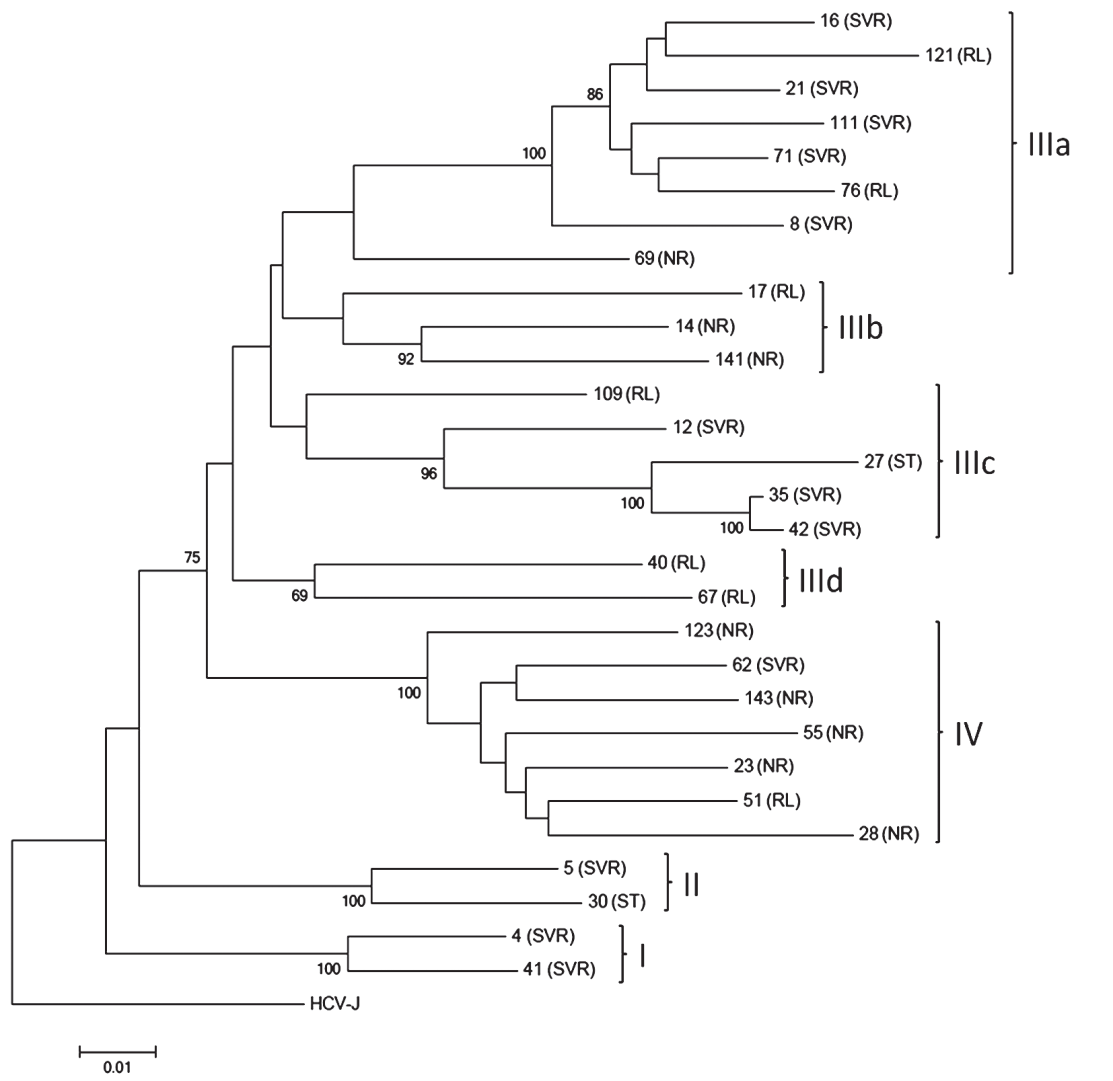
Phylogenetic Tree Illustrating Full-Length NS5A Sequence Relationships Between HCV Strains From Patients With Different Treatment Outcome Phylogenetic groups are denoted by Roman numerals in the right-hand column. Abbreviations: SVR, sustained virological response; RL, relapse; NR, non-response; ST, stopped treatment.

Twenty-nine full-length NS5A nt sequences were used for construction of a phylogenetic tree. At phylogenetic analysis isolated strains were segregated into four groups mainly according to treatment outcome (Figure 4). 

Thus, groups I and II were represented mainly by sequences from SVR patients. The most polymorphous group III was subdivided into four sub-groups. Sub-groups IIIb and IIId were formed by sequences obtained from only non-SVR patients, while sub-groups IIIa and IIIc were predominantly included sequences from SVR patients (8/12, 62%). In contrast, group IV was represented by 86% (6/7) of sequences obtained from non-SVR patients. Both samples from ST patients clustered with samples from SVR patients within group II and sub-group IIIc.

Considering the results of phylogenetic analysis, 24 group-specific nt positions were identified (Table 2). Thirteen nt mutations were silent, but eleven nt mutations caused group-specific aa polymorphisms (Table 3). Three of these were found in the V3 region, one in the NLS, one in the CRS region, and six were in the non-classified region. 

**Table 2.** NS5A Nucleotide Polymorphisms of Different Phylogenetic Groups of HCV 1b

Nt^a^Position	*6266*	*6341*	*6359*	*6392*	6493	*6503*	6648	6831	6832	6840	*6884*	6889
**HCV-J**	T	G	A	G	C	A	A	G	T	C	G	A
**I – SVR**^**a**^	T/C : 1/1^b^	G	G	A	C	A	A	A^c^	C^c^	C	G	A
**II - SVR**	T	A^c^	A	A	C	G^c^	A/G : 1/1	G	T	C	G	A
**IIIa,c – SVR**	T/C : 12/1	G	A/G : 12/1	A	C	A	A	G	T	C/G : 12/1	G	A
**IIIb – NR**^**a**^	T	G	A	A	C	A	A	G	T	C	G	A
**IIId – RL**^**a**^	T	G	A	A	T^d^	A	A	G	T	C	G	A
**IV - NR**	G^c^	G	C/T : 6/1^c^	G^c^	C/T : 6/1	A	G/T : 6/1^d^	G	T	G^d^	A^c^	G/A : 6/1^d^
	аа 7^a, e^				aa 83^e^		aa 135^e^	aa 196^e^		aa 199^e^		aa 215^e^
**Nt position**	7165	7220	*7244*	7249	*7274*	7315	*7007*	7450	7452	7462	*7463*	7465
**HCV-J**	G	G	C	T	T	G	C	A	G	A	A	G
**I - SVR**	G	G	C	A^c^	A	G	C	A	G	A^c^	A^c^	A^c^
**II - SVR**	G	T^c^	A^c^	T	A	A^c^	C	A	G	G^c^	A^c^	G
**IIIa,c – SVR**	G	G	C	T	A	G	C	A	G/A : 11/2	G/A : 12/1	C	G
**IIIb - NR**	G	G	C	T	G^c^	G	C	A	A^d^	G/A : 1/2	C	G
**IIId - RL**	A^c^	G	C	T	A	G	C	A/C : 1/1	G/A : 1/1	G/A : 1/1	C	G
**IV - NR**	G	G	C/T : 6/1	T	A	G	T^c^	G^c^	G	G	C	G
	aa 307^e^					aa 357^e^			aa 403^e^	aa 406^e^		aa 407^e^

^a^Abbreviation: Nt, nucleotide; SVR, sustained virological response; RL, relapse; NR, non-response; aa, amino acid.

^b^T/С : 1/1 denotes nucleotide substitution at position *6266 *(by HCV-J), 1 sequence from group I with T and 1 sequence with C.

^c^most relevant positions.

^d^ putatively applicable positions.

^e^ Amino acid substitutions caused by the found polymorphism.

**Table 3.** NS5A Amino Acid Polymorphisms for Different Phylogenetic Groups of HCV 1b^a^

Aa^b^position	CRS^b^	2055	2107	2168	2171	2187	2279	NLS^b^	V3^b^		
	1979							2329	2375	2378	2379
**HCV-J**	D	T	T	V	L	K	R	R	D	K	G
**I – SVR** ^**b**^	D	T	T	T	L	K	R	R	D	**K**	**E**
**II – SVR**	D	T	T/A : 1/1^c^	V	L	K	R	**K**	D	**E**	G/R:1/1
**IIIa,c – SVR**	D	T	T/I : 12/1	V	L/V : 12/1	K	R	R	D/N:11/2	A/T:12/1	R
**IIIb – NR** ^**b**^	D	T	T	V	L	K	R	R	**N**	A/T:1/2	R
**IIId – RL** ^**b**^	D	**M**	T	V	L	K	**K**	R	D/S:1/1	A/T:1/1	R
**IV – NR**	**E**	**T/M:6/1**	**A/S: 6/1**	V	V	**R/K:6/1**	R	R	D	A	R

^a^Amino acid positions are numbered according to HCV-J. Phylogenetic groups are denoted by Roman numerals. The most relevant residues marked by bold-type.

^b^ Abbreviations: Aa, amino acid; CRS, cytoplasmatic retention signal; NLS, nuclear localization signal; V3, variable region 3; SVR, sustained virological response; RL, relapse; NR, non-response

^c^ T/A: 1/1 denotes substitution Thr/Ala at position 2107, 1 sequence from group II with Thr and 1 sequence with Ala.

## 5. Discussion

This is the first study investigating the NS5A genetic variability in relation to PegINFα/RBV treatment response in Estonian HCV-1b infected patients.

The previous studies suggested that number of aa mutations in the ISDR region of NS5A can affect the efficacy of IFN-based therapy in Japanese patients with HCV-1b chronic infection. Thus, patients infected with the wild type ISDR sequence (identical to the prototype Japanese HCV strain, HCV-J) did not respond to IFN-based therapy whereas patients infected with the mutant type, defined by four or more amino acid substitutions in this region, achieved SVR (14, 18). Mutations in the PKRBD, V3 region or its upstream region near the carboxy terminus of NS5A, recently referred to as the IRRDR were also found to be of importance for the outcome of combination treatment (10, 19). However, attempts to extend this approach to European patients have not been successful (16, 17). It can be explained either by the existence of a methodological bias concerning selection of patients and therapeutic regimens (20) or by genetic differences between virus strains circulating in different populations (21). However, there is still limited information available regarding the correlation of NS5A full-length gene polymorphisms with response to IFN-based therapy.

In our study, entire aa NS5A sequences obtained from 29 patients were compared to the reference HCV-J strain. We did not find any mutant viruses carrying four or more aa changes in the ISDR region. The distribution of wild type (0 mutations), intermediate type (1 to 3 mutations), and mutant type (> 4 mutations) ISDR strains (17.2%, 82.8% and 0%, respectively) was similar to that reported previously in European populations, 24.8%, 63.4% and 11.8%, respectively (22), as well as for a Tunisian population, 26.7%, 66.7%, and 7.7%, respectively (10). 

There was not any significant difference between HCV strains isolated from the SVR and non-SVR patients in terms of either the rate of random mutations within the ISDR, PKRBD, V3 or IRRDR regions, or the total number of mutations in the entire NS5A gene as it has been described for HCV-1b strains isolated from European patients previously (23).

Several studies showed a higher (more than 600,000 to 800,000 IU/mL) pre-treatment viral load in patients with the wild-type ISDR sequence compared to that in patients with mutant sequences (14, 22, 24). Despite none of our patients had been infected with ISDR mutant-type strains, we found that the presence of R at position 2283 correlated significantly with a lower viral load, while P at the same position correlated with higher HCV RNA concentrations. 

A number of studies have focused on the detection of possible correlation between EVR and sequence variations within different parts of the NS5A gene, especially within the V3 region and its surrounding sequences (15, 25). However, we did not find any difference in the number of mutations within the analyzed regions between cEVR and non-EVR patients and observed additionally that cEVR was not reliable marker for prognosis of treatment outcome in Estonian patients. 

Previously it has been proposed that patients infected with HCV-1b who had aa mutations in NS5A at positions 2209, 2216, 2217, 2218, 2227, 2360, and 2378 (according to the enumeration of the HCV-J prototype strain) achieve SVR more frequently than those without mutations at the above mentioned positions (11, 12, 26). Recently three aa substitutions as I2268V, R2260H and S2278T were associated with unfavorable treatment outcome in HCV-1b patients in Hong Kong (21). We did not find any differences between the sequences obtained from SVR and non-SVR patients at mentioned above aa positions. However, we found that T1989 and R2283 correlated significantly with SVR; E1979, A2107, V2171, and A2382 were associated with non-response to treatment and aa substitution T2319A correlated with treatment relapse.

Some research groups used phylogenetic analyses of the complete NS5A sequence to predict treatment outcome, but failed to show any clustering associated with a specific pattern of response ( 27 ). In the present study 29 complete NS5A sequences were tentatively subdivided into several viral lineages within the HCV 1b subgenotype. Each lineage was characterized by one or more of the 24 novel nt polymorphisms which corresponded to 11 novel aa substitutions in the full-length NS5A protein. These substitutions may serve as tags to allow allocation of HCV 1b isolates from Estonia to one of the four structurally distinguishable phylogenetic clades characterized by different treatment response (Table 3). 

The present study has several limitations. One of the limitations of this study might be the selection bias arising from the National Guidelines for treatment of chronic hepatitis C. Another limitation is that the number of patients selected for the study was smaller than in most previous treatment studies in which more patients were enrolled. Further studies on a larger data set are needed to validate the significance of these found and other amino acid substitutions reported previously in relation to treatment response.

In conclusion, we have found a number of amino acid substitutions specific for HCV-1b strains isolated from patients with different treatment outcomes. Our results suggest the existence of HCV genetic lineages which are characterized by different risks for non-response outcome. However, some of these lineages may be prevalent only in small local populations.
